# Supplementary material for: Capturing Structural Variants of Herpes Simplex Virus Genome in Full Length by Oxford Nanopore Sequencing
Source: Microbiol Spectr. 2022 Aug 30;10(5):e02285-22. doi: 10.1128/spectrum.02285-22 (PMC9602439; doi:10.1128/spectrum.02285-22)
Supplement: Supplemental file 1 — Supplemental material. Download spectrum.02285-22-s0001.pdf, PDF file, 1.7 MB [file spectrum.02285-22-s0001.pdf]

Supplementary figure

Figure S1. Annotated full length/nearly full-length reads of Human herpes simplex virus 1 (HSV-1) isolate B^{3}x1.1 genome

A) Prototype (P) isomer

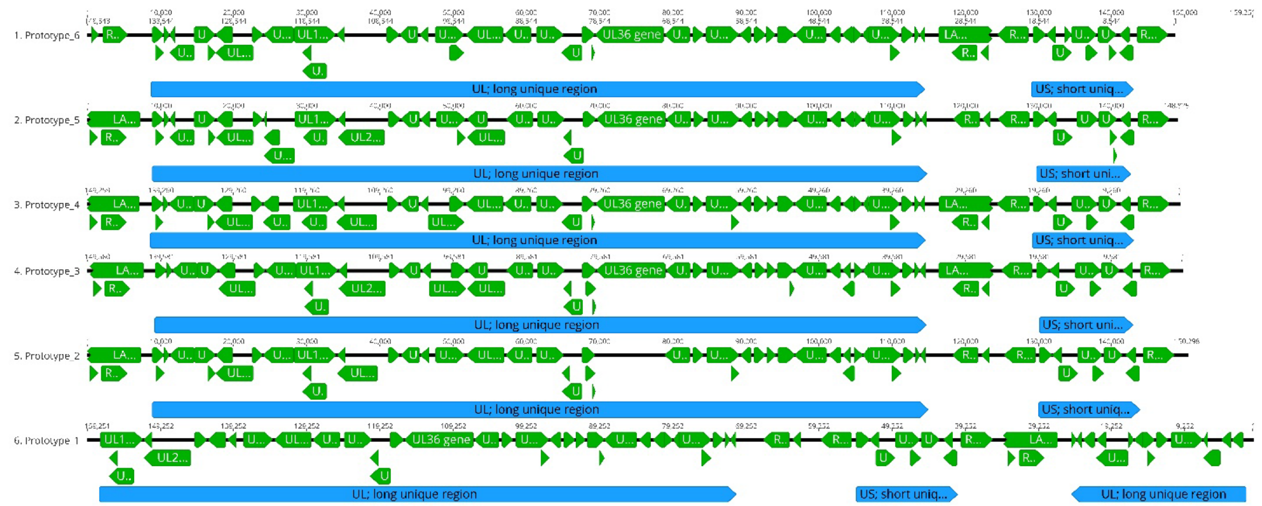

B) Inverted long (I_L) isomer

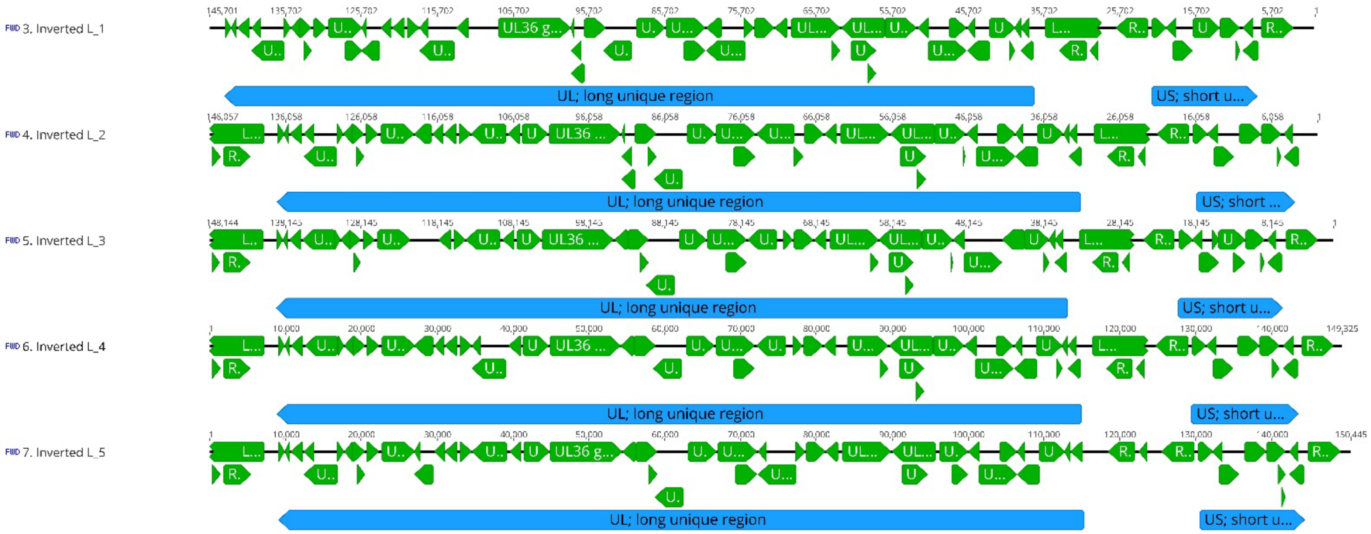

C) Inverted short (I_S) isomer

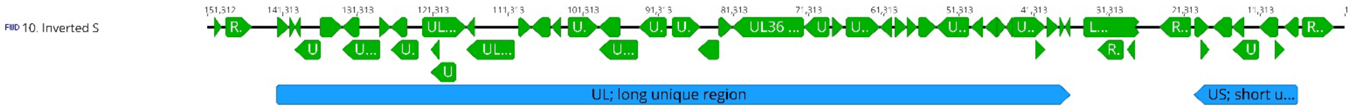

D) Inverted long and short (I_{LS}) isomer

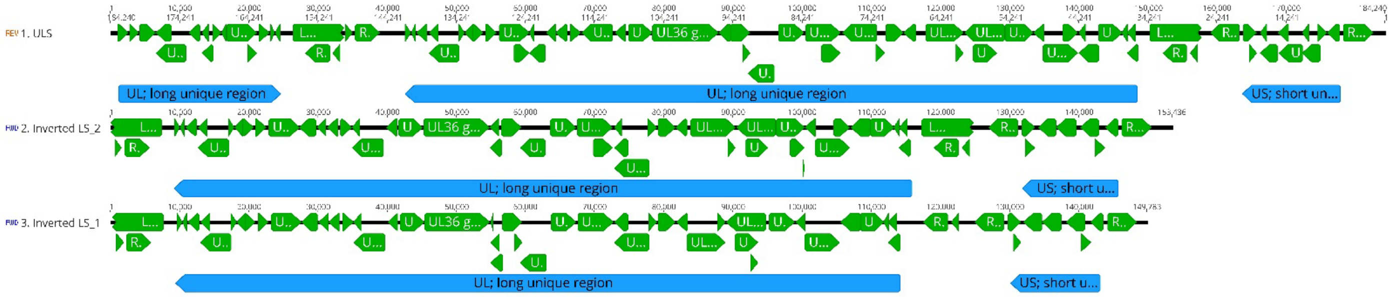

Figure S1. Oxford Nanopore sequencing captured 15 ultra-long reads with sizes ranging from 145 Kb to 184 Kb (6 Prototype, 5 I_L, 3 I_{LS} and one I_S). All full-length/nearly full-length reads were annotated by Geneious prime software (version 2022.0.1) using HSV-1 strain 17 as a reference.
